# The Detection and Sequencing of a Broad-Host-Range Conjugative IncP-1β Plasmid in an Epidemic Strain of *Mycobacterium abscessus* subsp. *bolletii*

**DOI:** 10.1371/journal.pone.0060746

**Published:** 2013-04-02

**Authors:** Sylvia Cardoso Leão, Cristianne Kayoko Matsumoto, Adriana Carneiro, Rommel Thiago Ramos, Christiane Lourenço Nogueira, James Daltro Lima Junior, Karla Valéria Lima, Maria Luiza Lopes, Horacio Schneider, Vasco Ariston Azevedo, Artur da Costa da Silva

**Affiliations:** 1 Disciplina de Microbiologia, Departamento de Microbiologia, Imunologia e Parasitologia da Escola Paulista de Medicina, Universidade Federal de São Paulo, São Paulo, SP, Brasil; 2 Laboratório de Polimorfismo de DNA, Instituto de Ciências Biológicas, Universidade Federal do Pará, Belém, PA, Brasil; 3 Seção de Bacteriologia e Micologia, Instituto Evandro Chagas, Ananindeua, PA, Brasil; 4 Instituto de Estudos Costeiros, Universidade Federal do Pará, Bragança, PA, Brasil; 5 Instituto de Ciências Biológicas, Universidade Federal de Minas Gerais, Belo Horizonte, MG, Brasil; University of Hyderabad, India

## Abstract

**Background:**

An extended outbreak of mycobacterial surgical infections occurred in Brazil during 2004–2008. Most infections were caused by a single strain of *Mycobacterium abscessus* subsp. *bolletii*, which was characterized by a specific *rpoB* sequevar and two highly similar pulsed-field gel electrophoresis (PFGE) patterns differentiated by the presence of a ∼50 kb band. The nature of this band was investigated.

**Methodology/Principal Findings:**

Genomic sequencing of the prototype outbreak isolate INCQS 00594 using the SOLiD platform demonstrated the presence of a 56,264-bp circular plasmid, designated pMAB01. Identity matrices, genetic distances and phylogeny analyses indicated that pMAB01 belongs to the broad-host-range plasmid subgroup IncP-1β and is highly related to BRA100, pJP4, pAKD33 and pB10. The presence of pMAB01-derived sequences in 41 *M. abscessus* subsp. *bolletii* isolates was evaluated using PCR, PFGE and Southern blot hybridization. Sixteen of the 41 isolates showed the presence of the plasmid. The plasmid was visualized as a ∼50-kb band using PFGE and Southern blot hybridization in 12 isolates. The remaining 25 isolates did not exhibit any evidence of this plasmid. The plasmid was successfully transferred to *Escherichia coli* by conjugation and transformation. Lateral transfer of pMAB01 to the high efficient plasmid transformation strain *Mycobacterium smegmatis* mc^2^155 could not be demonstrated.

**Conclusions/Significance:**

The occurrence of a broad-host-range IncP-1β plasmid in mycobacteria is reported for the first time. Thus, genetic exchange could result in the emergence of specific strains that might be better adapted to cause human disease.

## Introduction

An epidemic of surgical-site infections caused by a specific strain of rapidly growing mycobacteria (RGM) affected thousands of patients submitted to laparoscopic, arthroscopic and plastic surgeries in several states of Brazil between 2004 and 2008 [1], [2], [3], [4], [5]. A single strain of *Mycobacterium abscessus* subsp. *bolletii*, which was characterized by a specific *rpoB* sequevar (accession number EU117207) and two closely related pulsed field gel electrophoresis (PFGE) patterns, was responsible for these infections [4]. This strain was susceptible to amikacin and clarithromycin and resistant to tobramycin, ciprofloxacin, sulfamethoxazole, minocycline and doxycycline [6], [7], [8]. The strain was highly resistant to glutaraldehyde and susceptible to orthophthaldehyde and peracetic acid-based solutions [3], [9]. One isolate was deposited in the collection of the Brazilian National Institute for Health Quality Control (INCQS) under accession number INCQS 00594.

The molecular typing of 152 isolates using PFGE revealed two highly similar patterns. When the DNA was digested with *Dra*I or *Ase*I, a majority of the isolates (124/152) showed a ∼50-kb band, while 28 isolates lacked this band. These results were highly reproducible, although in particular cases the ∼50-kb band was clearly visible on the first PFGE gel but was absent in subsequent gels performed with isolated colonies. Occasionally, the opposite effect was also observed. These observations suggested that the ∼50-kb band was an extra-chromosomal plasmid. Linear [10], [11], [12] and circular plasmids [13], [14], [15], [16] were recently reported in mycobacteria.

IncP-1 plasmids have not been previously described in mycobacteria. These plasmids were first detected in *Pseudomonas aeruginosa* and enterobacteria from clinical and non-clinical sources, such as water, activated sludge and soil, and different hosts, some of which are unknown [17]. Five IncP-1 plasmid subgroups (α, β, γ, δ and ε) have been defined based on the phylogenetic relatedness of the TrfA proteins and plasmid organization. Complete genomic sequences of representatives of IncP-1α (e.g., RP4 [18]), IncP-1β (R751 [19], pB4 [20] and pB10 [21]), IncP-1γ (pQKH54 [22]), IncP-1δ (pEST4011 [23] and pAKD4 [24]) and IncP1-ε (pKJK5 [25]) are available. These plasmids are important mediators of horizontal gene transfer, particularly for the transfer of antibiotic resistance determinants and genes responsible for the degradation of complex compounds and xenobiotics [26], [27]. These genes are usually located in genetic load regions that interrupt the conserved backbone and are used for the delineation of these plasmids in phylogenetically distinguishable lineages.

Here we describe the complete DNA sequence and genetic organization of an IncP-1β circular 56,264-bp plasmid present in the epidemic strain INCQS 00594 and its distribution in other *M. abscessus* subsp. *bolletii* isolates. Experiments to obtain transconjugants and transformants with this plasmid in *Escherichia coli* and *Mycobacterium smegmatis* mc^2^155 were performed.

## Results

### Sequencing and assembly of pMAB01

We obtained the whole genomic sequence of the prototype strain INCQS 00594 and identified sequences that matched the plasmid sequences deposited in the GenBank database.

Genomic sequencing of the outbreak strain prototype isolate INCQS 00594 using the SOLiD V3 platform (Life Technologies, CA, USA) generated 72,195,458 reads. A total of 33,406,401 reads remained after the application of the Phred 20 quality filter and were used for *ab initio* assembly. The number of contigs obtained with an N50 of 380 bp was 9,898, the longest of which was 2,271 bp, for a total of 3,625,660 bp.

A BLAST evaluation against the non-redundant nucleotide databank (NT) from NCBI identified 117 contigs that matched to sequences in plasmid pB10 (accession number: NC_004840) with an e*-value* of *<*1×10^5^. This plasmid was therefore used as a reference to map the filtered short reads to generate a 56,252**-**bp scaffold.

A low-coverage region, showing conflicts in the reads between positions 43,732 and 49,025, was sequenced using the Sanger method with the primers shown in Table 1, resulting in the identification of a 5,294-bp consensus fragment. The fragment was aligned to the sequences flanking the low-coverage region, and the bases were incorporated into the plasmid sequence. The region between positions 17,269 and 19,241 was also sequenced using primers derived from the obtained sequence and plasmid pB10 (Table 1). Using this procedure, 12 new bases that were previously unidentified in the assembly step were added to the sequence, resulting in a final consensus scaffold of 56,264 bp. The plasmid was named pMAB01.

**Table 1:** Primers used for the amplification and sequencing of the transposon (A) and integron (B) regions using the Sanger method and the amplification of selected pMAB01 genes (*trfA* to *merE*).

Region	Primer	Sequence (5' – 3')	Primer position in the pMAB01 sequence	Amplicon size (bp)	Function
A	pMAB01-1F	GTGCGCCTCGGCTCTCACTCC	43,732 – 43,752	608	
	pMAB01-583R	TTATCCATTGACACTTGAGGG	44,319 – 44,339		
	pMAB01-507F	GTTAAAAACCTGGTTAGCATG	44,243 – 44,263	506	
	pMAB01-990R	GCACTGAAAACGAGGAGACCC	44,728 – 44,748		
	pMAB01-850F	AAGTGTCAACGTCAGGGCTGC	44,586 – 44,606	720	
	pMAB01-1547R	TGAGGCTGGCGTGCAATTGGG	45,285 – 45,305		
	pMAB01-1318F	CATCTGTGAGGTATTCCACGC	45,056 – 45,076	664	
	pMAB01-1961R	CTACTCCCATGTAAAAGACCG	45,699 – 45,719		
	pMAB01-1879F	TTCTTGGCGGATGACACCTTC	45,617 – 45,637	584	
	pMAB01-2442R	ATGATGAACTCCATTTGTCGC	46,180 – 46,200		
	pMAB01-2301F	CACGTCCGAGCATGATCCAGC	46,039 – 46,059	705	
	pMAB01-2985R	AAGCCTTGATCACCGAAACCC	46,723 – 46,743		
	pMAB01-2838F	GCTTGGCAAGGTACTCGATGG	46,576 – 46,596	994	
	pMAB01-3408R	TTGTGGCGCCACCTTGGCAAC	47,549 – 47,569		
	pMAB01-3326F	CTTGTTGGTGATCGAATAGCG	47,467 – 47,487	730	
	pMAB01-3926R	GCTTTGTGCCGCGGCTCTCGT	48,176 – 48,196		
	pMAB01-3778F	CGAGGAGACCCCGAGTCACCC	47,919 – 47,939	765	
	pMAB01-4413R	CGCGTCGCGTCACTCTAACCG	48,663 – 48,683		
	pMAB01-4338F	CATCACTCAGCGTATAGTGCT	48,588 – 48,608	438	
	pMAB01-4764R	ACAAAATCACCAGATTCTCCG	49,005 – 49,025		
B	pMAB01-17248F	ATCTGATCGGACAGGGCGTCT	17,268 – 17,288	524	
	pMAB01-17737R	AATCGCAACATCCGCATTAAA	17,771 – 17,791		
	pMAB01-17874F	TTTGGCTGTGAGCAATTATGT	17,908 – 17,928	1,334	
	pMAB01-18173R	GCAACTGGTCCAGAACCTTGA	19,221 – 19,241		
	pB10-16895F	ACGATAGAGCTTCCTGAGAAA	16,895 – 16,915 *	217	
	pB10-17091R	TCAAGACCAAGATTTGCGATC	17,091 – 17,111 *		
*trfA*	trfA1-F	AGTGCGATGGCGACCAAGAAG	841 – 861	446	replication
	trfA1-R	AGGCATTCCTCGGCCCTTGTG	416 – 436		initiation
*ssB1*	ssB1-F	GCCGTTTTCGACCTGGCTGTC	1,511 – 1,531	230	ssDNA
	ssB1-R	TATTCGATTTCCTCGGCGATG	1,302 – 1,322		binding
*trbE*	trbE-F	CCTGAGCTGGTTGCAGTTCTG	4,839 – 4,859	453	mating pair
	trbE-R	AGCACGACTACGCTGGTGTAG	5,271 – 5,291		formation
*sul1*	sul1-F	TTCCTGACCCTGCGCTCTATC	17,158 – 17,178	369	sulfonamide
	sul1-R	CTGGACCCAGATCCTTTACAG	16,810 – 16,830		resistance
*qacEdelta1*	qacEdelta-F	GCAATAGTTGGCGAAGTAATC	17,788 – 17,808	272	multidrug
	qacEdelta-R	AGCAAAAAGGCAGCAATTATG	17,537 – 17,557		efflux protein
*traE*	traE-F	TTCTACCCGCTGCACGAGTAC	26,477 – 26,497	701	conjugative
	traE-R	TTGCTCGGCCGGCGCGACAAG	25,797 – 25,817		DNA transfer
*oriT*	oriT - F	TGCCTCGCAGAGCAGGATGAC	32,882 – 32,902	287	origin of
	oriT - R	GCTACGGAAACGCAAAAAGTC	33,148 – 33,168		transference
*kleE*	kleE-F	ATCAAGTTCCCCAAGGAGATC	40,243 – 40,263	299	stable
	kleE-R	TTGTAGATCGAAACGAAGTAG	39,965 – 39,985		inheritance
*strA*	strA-F	GCGGAGAATCTGGTGATTTTG	49,004 – 49,024	361	streptomycin
	strA-R	TTGCGGGACACCACATCAACG	49,344 – 49,364		resistance
*merE*	merE-F	ATGAACAACCCCGAGCGCTTG	52,851 – 52,871	204	mercury
	merE-R	CGACAGGGACAGAAGGAACAG	52,668 – 52,688		resistance

* : Primer position in the pB10 plasmid sequence (GenBank accession number NC_004840)

### The backbone structure and comparative analysis

The automatic annotation using RAST led to the prediction of 64 coding sequences (CDS) and 2 pseudogenes. The open reading frames (ORFs) showed 99 – 100% identity with homologous sequences from IncP-1β subgroup plasmids according to a BLASTP analysis against the NR databank. A total of 44 CDS showed best hits with plasmid pB10, and the remaining 20 showed best hits with the sequences from plasmids R751, R100, pTET3, pJP4, BRA100, pCTX-M3, pRAx, pADP-1, pIG1, RSF1010 and JMP134 (Table 2). The pMAB01 genome revealed a 64.27% guanine and cytosine (G+C) content, which is similar to the content of other IncP-1 plasmids, and each CDS G+C frequency showed a uniform distribution with small variations throughout the genome. The ORFs *qacEdelta1, aac(6')-Ib* and pMAB01_024 showed the lowest G+C values.

**Table 2:** Predicted coding sequences on plasmid pMAB01 and the putative functions.

Coding sequence	Protein size (aa)	GC content (%)	Putative function - best hit homolog in the database*	GenBank accession no. of the best hit	Identity to the closest homolog in the database
*trfA1*	406	66	Replication initiation protein - plasmid pB10	NP_858039	100
*ssb*	113	60	Single-stranded DNA-binding protein - plasmid R751	NP_044238	100
*trbA*	120	59	Conjugal transfer protein TrbA - plasmid pB10	NP_857976	100
*trbB*	320	62	Conjugal transfer protein trbB - plasmid pB10	NP_857977	100
*trbC*	154	67	Conjugal transfer protein TrbC - plasmid pB10	NP_857978	100
*trbD*	103	63	Conjugal transfer protein TrbD - plasmid pB10	NP_857979	100
*trbE*	852	64	Conjugal transfer protein TrbE - plasmid pB10	NP_857980	100
*trbF*	260	66	Conjugal transfer protein TrbF - plasmid pB10	NP_857981	100
*trbG*	306	64	Conjugal transfer protein TrbG - plasmid pB10	NP_857982	100
*trbH*	162	71	Conjugal transfer protein TrbH - plasmid pB10	NP_857983	100
*trbI*	473	67	Conjugal transfer protein TrbI - plasmid pB10	NP_857984	100
*trbJ*	254	61	Conjugal transfer protein TrbJ - plasmid R751	NP_044248	100
*trbK*	75	62	Conjugal transfer entry exclusion protein TrbK - plasmid pB10	NP_857986	100
*trbL*	571	68	Conjugal transfer protein TrbL - plasmid pB10	NP_857987	100
*trbM*	195	68	Conjugal transfer protein TrbM *-* plasmid pB10	NP_857988	100
*trbN*	211	69	Conjugal transfer protein TrbN - plasmid pB10	NP_857989	100
*trbO*	88	64	Conjugal transfer protein TrbO - plasmid R751	NP_044253	100
*trbP*	232	68	Conjugal transfer protein TrbP - plasmid pB10	NP_857991	100
pMAB01_019	143	66	Outer membrane protein precursor - plasmid pB10	NP_857992	100
orf-5	166	65	GCN5-like N-acetyltransferase - plasmid R100	NP_052894	100
*sulI*	279	62	Dihydropteroate synthase type-1 - plasmid pTET3	NP_478074	100
*qacEdelta1*	115	50	Ethidium bromide resistance protein - plasmid R100	NP_052896	100
*aac(6')-Ib*	172	54	Aminoglycoside 6'-N-acetyltransferase - plasmid BRA100	YP_006316013	100
pMAB01_024	134	54	Glyoxalase-like domain protein - plasmid BRA100	YP_006316014	100
*intI*	337	61	Class I integron integrase - plasmid pCTX-M3	NP_775042	100
pMAB01_026	136	65	Hypothetical protein - protein pRAx	YP_002995716	100
*traC*	1448	69	DNA primase TraC - plasmid pB10	NP_857999	100
*traD*	129	73	Protein TraD - plasmid R751	NP_044268	100
*traE*	687	67	DNA topoisomerase III - plasmid pB10	NP_858001	100
*traF*	178	66	Plasmid transfer protein TraF - plasmid R751	NP_044270	100
*traG*	637	66	Conjugal transfer protein TraG - plasmid pB10	NP_858003	100
*traI*	746	67	DNA relaxase - plasmid pB10	NP_858004	100
*traH*	130	68	Relaxosome stabilizing protein - plasmid R751	NP_044273	100
*traJ*	124	67	Conjugal transfer relaxosome component TraJ - plasmid pB10	NP_858006	100
*traK*	132	63	Conjugal transfer protein TraK - plasmid R751	NP_044276	100
*traL*	241	63	Conjugal transfer protein TraL- plasmid pB10	NP_858008	100
*traM*	146	68	Conjugal transfer protein TraM - plasmid R751	NP_044278	100
*traN*	217	69	Protein TraN - plasmid pB10	NP_858010	100
*traO*	115	58	Protein TraO - plasmid pB10	NP_858011	100
*kfrA*	343	75	Protein KfrA - plasmid pB10	NP_858012	100
*korB*	349	67	Transcriptional repressor protein KorB - plasmid pB10	NP_858013	100
*incC2*	254	65	Inclusion membrane protein IncC2 - plasmid pB10	NP_858015	100
*incC1*	360	65	Inclusion membrane protein IncC1 - plasmid pB10	NP_858014	100
*korA*	102	63	Transcriptional repressor protein KorA - plasmid pB10	NP_858016	100
*kleF*	109	63	Stable inheritance protein KleF - plasmid pB10	NP_858017	100
*kleE*	108	63	Stable inheritance protein KleE - plasmid pB10	NP_858018	100
*kleA*	78	67	Stable inheritance protein KleA - plasmid pB10	NP_858019	100
*korC*	85	68	Transcriptional repressor protein KorC - plasmid pB10	NP_858020	100
*klcB*	402	70	Stable inheritance protein KlcB - plasmid pB10	NP_858021	100
pMAB01_050	87	68	Hypothetical protein - plasmid pJP4	YP_293608	100
*klcA*	142	68	Antirestriction protein KlcA - plasmid pB10	NP_858022	100
*din*	87	65	Damage inducible-like protein - plasmid pB10	NP_858023	100
*relE*	93	68	Plasmid stabilization system - plasmid pB10	NP_858024	100
*tnpA*	971	60	Transposase for insertion sequence element IS*1071* in transposon Tn5271 - plasmid pADP-1	NP_862478	100
*strA*	267	56	Aminoglycoside resistance protein A - plasmid pIG1	NP_054472	100
*strB*	278	56	Aminoglycoside resistance protein B - plasmid RSF1010	NP_044302	100
*tnpR*	186	62	Transposon Tn21 resolvase - plasmid pB10	NP_858031	100
orf-2	329	62	Diguanylate phosphodiesterase - plasmid pB10	NP_858032	100
*merE*	78	65	Mercuric resistance protein MerE - plasmid pB10	NP_858033	100
*merD*	121	70	HTH-type transcriptional regulator MerD - plasmid pB10	NP_858034	100
*merA*	561	66	Mercuric reductase - plasmid pB10	NP_858035	100
*merP*	91	62	Mercuric transport protein periplasmic component - plasmid pB10	NP_858036	100
*merT*	116	62	Mercuric transport protein - plasmid pJP4	YP_025416	100
*merR*	144	61	Mercuric resistance operon regulatory protein - plasmid pB10	NP_858038	100

* : Certain CDSs showed more than one best hit, although the first one was selected to represent the homologous sequence.

**Figure 1:**
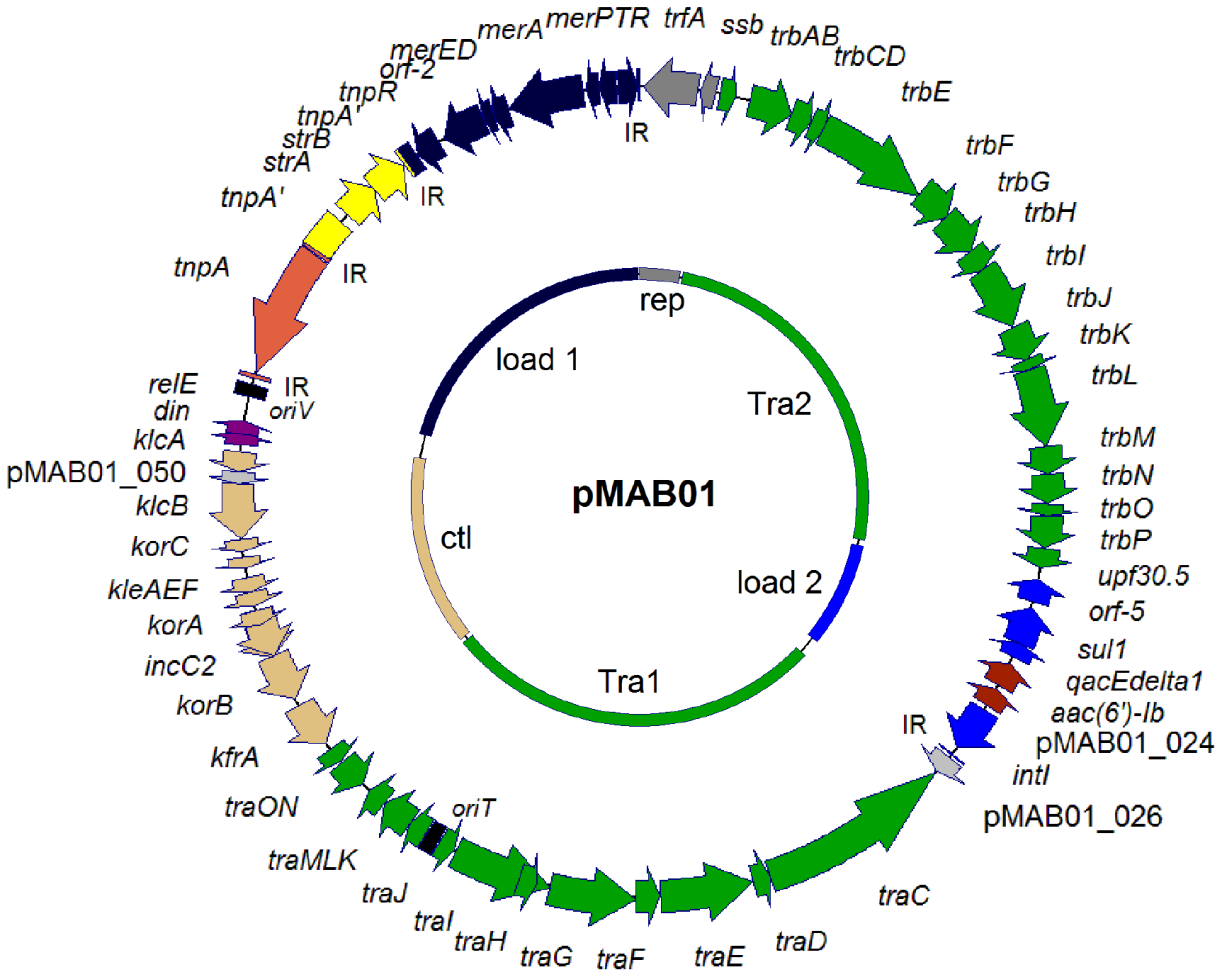
Genetic map of the IncP-1β plasmid pMAB01. The coding regions are indicated with arrows showing the direction of transcription. The inverted repeats (IRs) of the transposons and integrons are identified. The origins of vegetative replication (*oriV*) and plasmid transfer (*oriT*) are indicated with black boxes. The functional modules of the plasmid backbone are differentiated by colors in the inner circle: Tra1 (*tra*) and Tra2 (*trb*) (green); replication (rep) module (*trfA-ssb*) (grey); central control (ctl) region encoding regulatory and stability functions (*kfrA – relE*) (light brown). One genetic load region (load 1) contains the following: a Tn*501*-like mercury-resistance (*mer*) transposon (dark blue); a truncated Tn*5393c* streptomycin-resistance transposon (yellow); and a copy of the insertion element IS*1071* (orange). The second genetic load region (load 2) contains a class 1 integron with an integrase (*intI*) and the integron-specific segment *qacEdelta1-sul1-orf5* (light blue), with two cassettes, encoding an aminoglycoside 6'-N-acetyltransferase (*aac(6’)-Ib*) and a glyoxalase-like domain protein (pMAB01-024) (brown). A detailed description of the accessory regions is shown in Figure 2.

The overall structure and genetic organization of pMAB01 were similar to the backbones of self-transmissible and promiscuous IncP-1 plasmids, such as pB4 [20], pB10 [21], R751 [19] and pAKD33 [24]. pMAB01 contained four functional gene clusters that are characteristic of IncP-1 plasmids from subgroups α and β: (1) the Tra2 group of mating pair formation genes (*trbABCDEFGHIJKLMNOP*), (2) the Tra1 group of conjugative transfer genes (*traCDEFGHIJKLMNO*), (3) genes for plasmid maintenance, partitioning and control (*kfrA, korABC, incC, kleAEF, klcAB*) and (4) replication genes (*trfA, ssb*) and *oriV* containing eight direct repeats called iterons (5’TGACACTTGAGGGGC3’), which bind to TrfA to initiate replication [18] (Figure 1).

**Figure 2:**
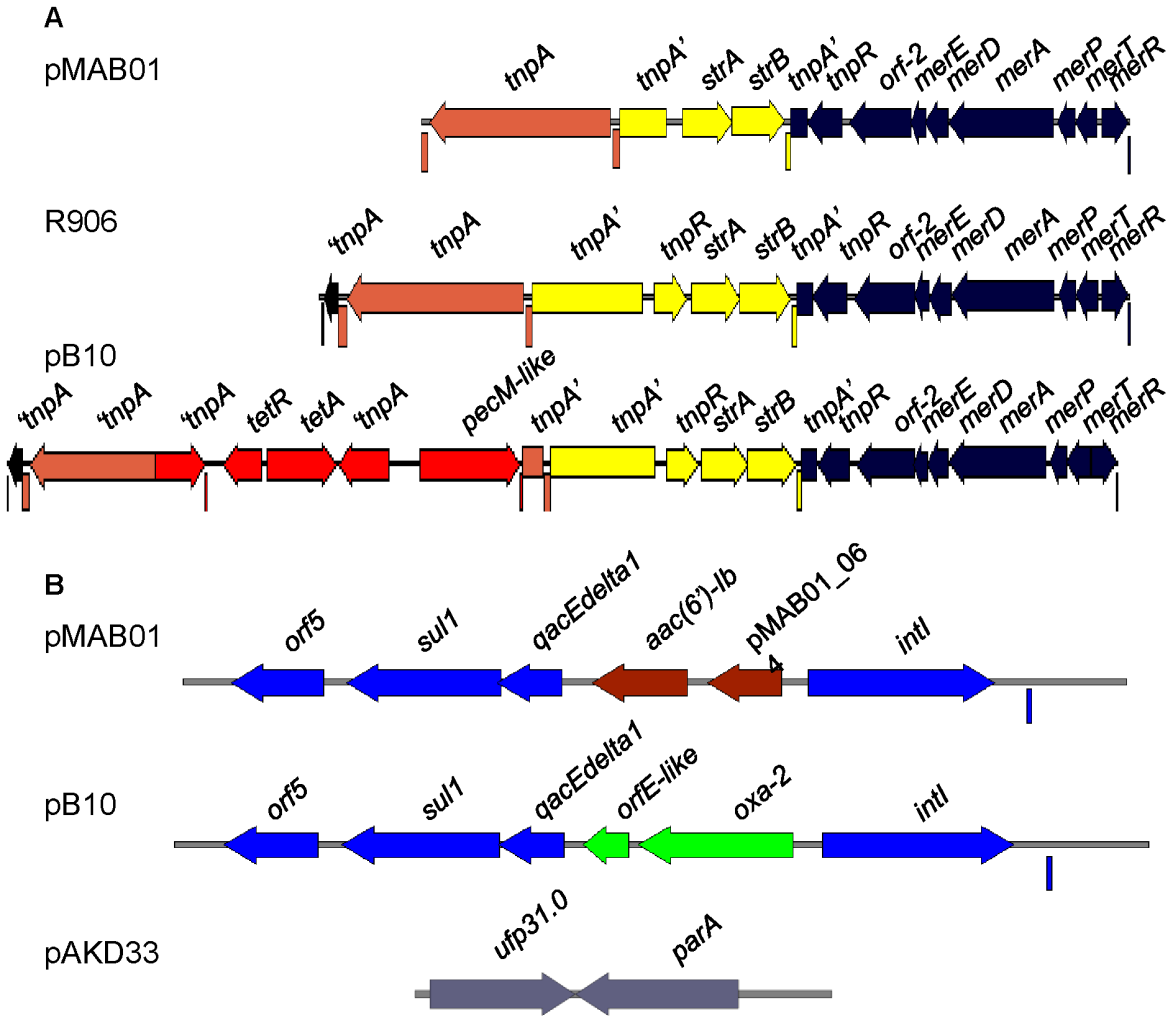
Schematic representation of the genetic load regions of IncP-1β plasmids. (**A)** Genetic load region downstream of the gene *trfA* from plasmids pMAB01, R906 and pB10. The transposable elements and corresponding IR sequences (rectangles below each map) are shown in different colors: Tn*501*-like mercury-resistance (*mer*) transposon (dark blue); Tn*5393c* streptomycin-resistance transposon (yellow); insertion sequence element IS*1072* (orange); Tn*1721* tetracycline-resistance transposon (red). The arrows indicate the direction of transcription. The designation ‘tnpA indicates a 3’ section of a truncated *tnpA* transposase gene, whereas tnpA’ indicates the 5’ region of a truncated *tnpA* transposase gene. (**B)** A comparison between the genes of the Tra1 and Tra2 regions from pMAB01, pB10 and pAKD33. The integrons in pMAB01 and pB10 contain the integrase gene (*intI*), a small multidrug exporter protein gene (*qacEdelta1*), a sulfonamide resistance gene (*sul1*) and a putative acetyltransferase (*orf5*) *(*light blue). The gene cassettes are different in pMAB01 (brown) and pB10 (green) (see text). In pAKD33, this region lacks accessory genes and contains the genes *ufp31.0* and *parA*, which enhance the stable inheritance of the plasmid via the resolution of multimers.

The plasmid pMAB01 was demonstrated to contain two genetic load regions (Figure 2). The first region is located in the origin of vegetative replication downstream of the replication initiation gene *trfA* and contains a Tn*501*-like class II mercury-resistance (*mer*) transposon. The transposase (*tnpA’*) gene of the Tn*501*-like transposon is interrupted after 264 bp by the insertion of a remnant copy of the Tn*5393c* streptomycin-resistance (*str*) transposon. This element has intact streptomycin-resistance genes (*strAB*), although there is a deletion of 547 bp in the region comprising the resolvase gene (*tnpR*) gene, and the 3’ end beyond 753 bp of the transposase gene (*tnpA’*) is missing. An intact copy of IS*1071* without direct repeats is present after the Tn*5393c* truncated transposon (Figure 2a).

The intergenic region between the conjugative transfer modules Tra1 and Tra2 contains a typical class 1 integron that is also present in plasmid pB10 (Figure 2b). This region encodes three groups of genes: (1) integrase (*intI*) followed by a recognition sequence (*attI*); (2) two incorporated gene cassettes encoding an aminoglycoside 6'-N-acetyltransferase (*aac(6’)-Ib*) and a hypothetical protein with a glyoxalase-like domain (pMAB01_024); and (3) a conserved 3′ segment containing a gene that encodes a determinant of ethidium bromide, the quaternary ammonium compound resistance gene (*qacEdelta1*), dihydropteroate synthase type-1, which confers sulfonamide resistance (*sul1*), and a conserved orf (*orf5*) that encodes a GCN5-like N-acetyltransferase.

### Genetic distance and phylogenetic analysis

**Figure 3:**
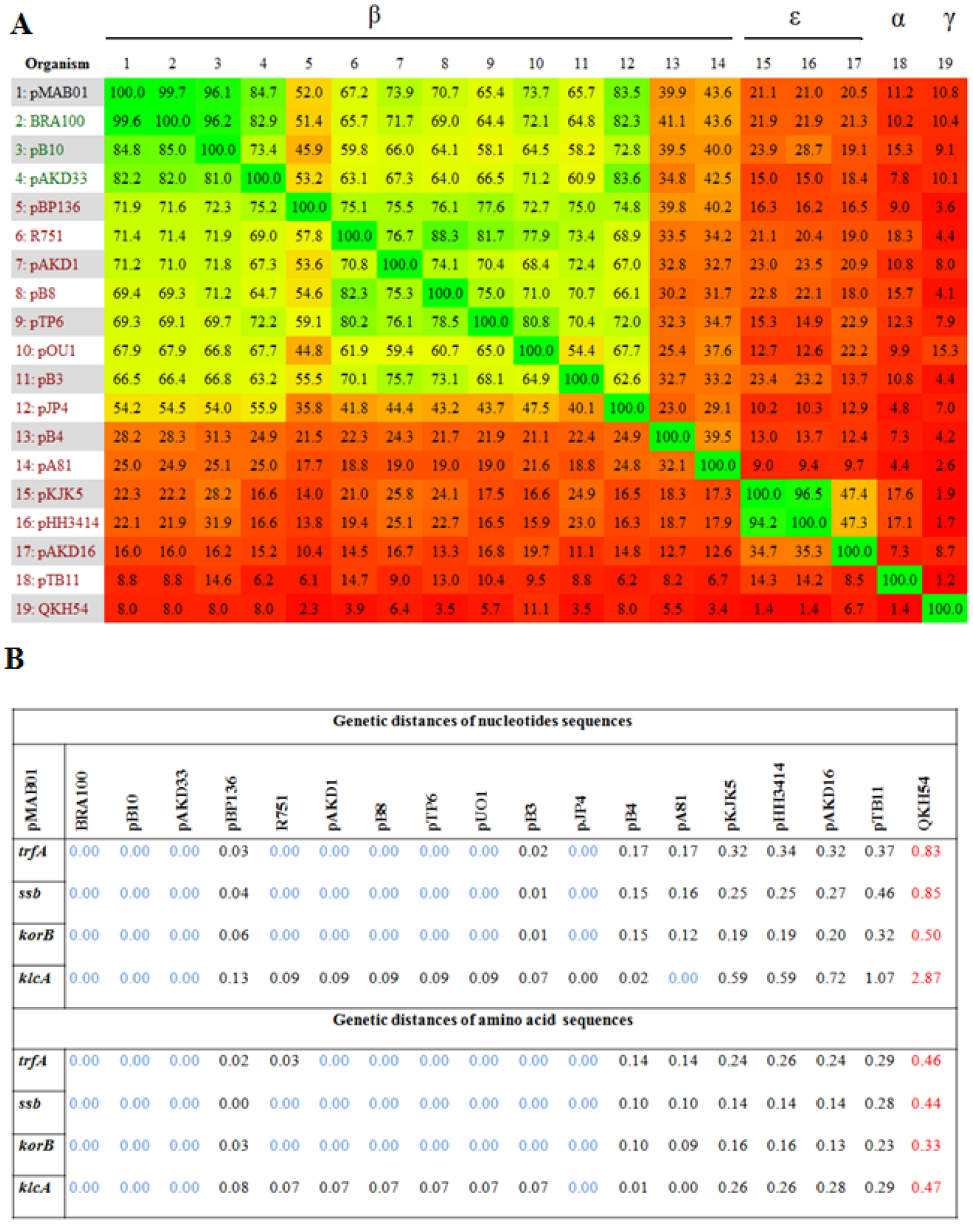
Heat-plot of the identity and genetic distance matrices between pMAB01 and the other 18 IncP-1 plasmid sequences. (**A)** Identity matrix generated from complete plasmid genome sequences; the heat-plot is based on a fragmented alignment constructed using BLASTN. (**B)** Genetic distance matrix obtained with nucleotide and amino acid sequences from genes *trfA, ssb, korB* and *klcA*; the plasmid with the greatest distance to pMAB01 is shown in red, and the smallest distances are shown in blue for each gene.

The identity matrix obtained from the phylogenetic analysis comparing the complete genome of plasmid pMAB01 with other IncP-1 plasmids showed that pMAB01 was highly related to the IncP-1β plasmids BRA100, pB10 and pAKD33 (Figure 3A), with values ranging from 84.7 to 99.7%. A comparison of selected nucleotide and amino acid sequences of pMAB01 and 18 IncP-1 plasmids indicated that pMAB01 showed the smallest genetic distances with IncP-1β plasmids BRA100, pB10, pAKD33 and pJP4 for all analyzed genes (Figure 3B). When considering the genes *trfA*, *ssb* and *klcA,* the IncP-1β plasmids pB3, pB4, pA81 and pBP136 showed larger genetic distances relative to plasmid pMAB01, and the *klcA* gene had the largest variability among all analyzed sequences. Plasmids pKJK5, pHH3414, pAKD16, pTB11 and QKH54, belonging to subgroups ε, α and γ, showed the largest distance values for all studied genes.

**Figure 4:**
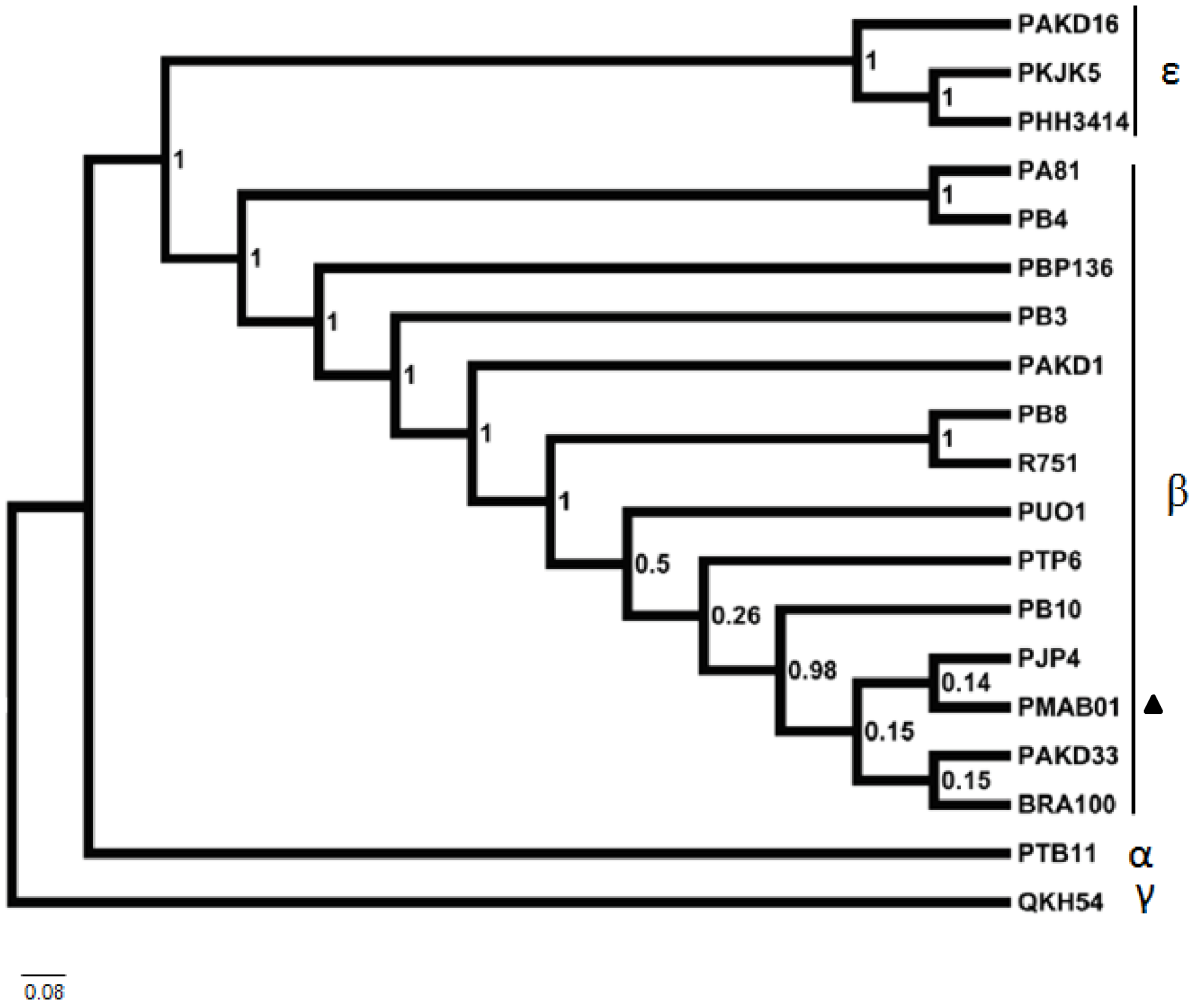
Phylogenetic tree of the genes *trfA*, *ssb*, *korB* and *klcA* from IncP-1 plasmids. The eighteen IncP-1 plasmid sequences corresponding to sub-groups α, β, γ and ε were obtained from GenBank, and the pMAB01 sequences were obtained in this study. The posterior credibility values are represented for each node. Plasmid QKH54 was used as outgroup. Plasmid pMAB01 is indicated with a black triangle. The scale bar corresponds to the nucleotide substitution rate. The vertical distance is provided for illustrative purposes only.

Based on the posterior credibility values (Figure 4), the majority of the nodes of the Bayesian phylogenetic tree were well supported. The sequencing results showed that pMAB01 is part of a group comprising plasmids pB10, pJP4, pMB01, pAKD33 and BRA100, and this association was supported by significant posterior credibility values (98%). The results of the Bayesian analysis also showed that the archetype IncP-1 plasmid R751 is closely related to pB8 (100%) and that pA81 is closely related to pB4 (100%). The plasmids pAKD16, pKJK5 and pHH3414 formed a strongly supported (100%) monophyletic group.

Overall, our results indicate that pMAB01 should be classified with the highly related IncP-1β group.

### Detection of plasmid sequences in *M. abscessus* subsp. *bolletii* isolates

**Figure 5:**
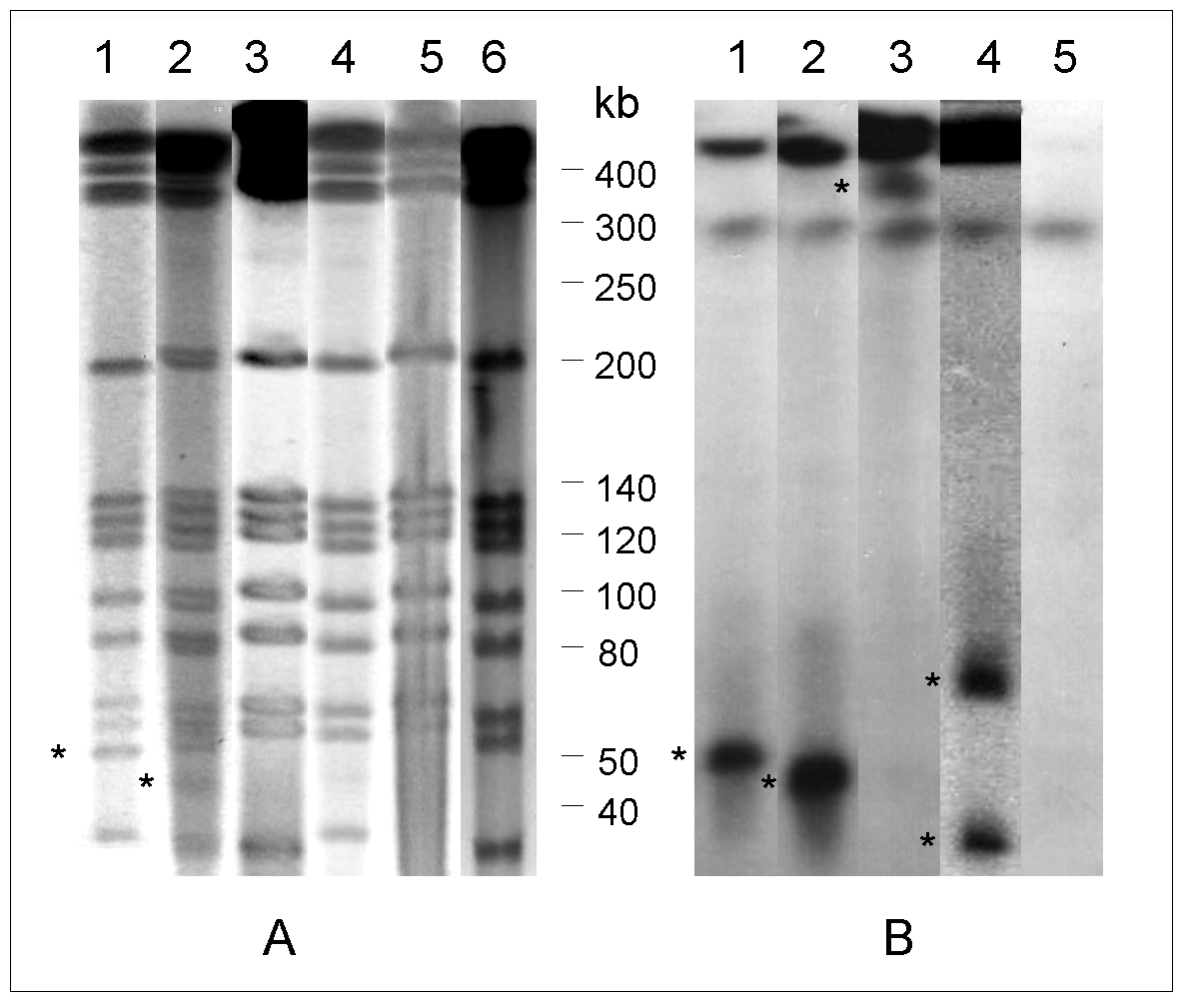
Pulsed field gel electrophoresis (PFGE) and Southern blot hybridization results. (**A)** PFGE-*Dra*I and (**B)** Southern blot hybridization using the *trfA*-derived probe of selected isolates showing the PFGE patterns of the epidemic strain: 1, the sequenced isolate INCQS 00594, showing the ∼50 bp PFGE-*Dra*I band that hybridized with the *trfA* derived probe; 2, isolate showing a band with faster migration in PFGE-*Dra*I and Southern blot hybridization; 3, no plasmid band detected in PFGE-*Dra*I and a hybridization band visible near gel origin; 4, two plasmid bands with different migration; 5, no evidence of the presence of pMAB01 either using PFGE-*Dra*I or hybridization; and 6, cured INCQS 00594 colony. The asterisks indicate the plasmid bands.

The presence of pMAB01 in 15 *M. abscessus* subsp. *bolletii* isolates obtained from surgical-site infections, 24 isolates from other specimens and two reference strains was verified using PCR with primers derived from ten different regions of pMAB01 and Southern blot hybridization using the *trfA* amplicon as a probe. The results of PCR, PFGE-*Dra*I and Southern blot hybridization are shown in Table 3 and Figure 5.

**Table 3:** Detection of pMAB01 plasmid sequences in *Mycobacterium abscessus* subsp. *bolletii* clinical isolates and type strains.

Isolates	N	PCR (pMAB01)	PFGE-*Dra*I (∼50 kb band)	PFGE-*Dra*I (< 50 kb band)	Hybridization (*trfA* probe)
Surgical isolates (epidemic	9	+	+		+
strain)	3	+		+	+
	1	+	–		+*
	2	–	–		–
Non-surgical isolates (epidemic	3	+	+		+
strain)	8	–	–		–
Other strains	13	–	–		ND
Type strains**	2	–	–		–
Total	41				

N: Number of isolates

+: Positive result

–: Negative result

ND: not determined

* : Near gel origin or between the 50 and 100-kb molecular markers (two different experiments with the same isolate)

** : *M. abscessus* ATCC 19977 and *M. massiliense* CCUG 48898

Fifteen epidemic isolates were subjected to PCR using the ten pMAB01-derived primer pairs shown in Table 1. Thirteen isolates produced amplicons with all primer pairs, and nine of these showed the ∼50-kb band in the PFGE-*Dra*I analysis and the corresponding hybridization band when using the *trfA1* probe (Figure 5 – lane 1). In the PFGE-*Dra*I and Southern blot hybridization analyses, three isolates showed a band that migrated below the 50-kb molecular marker (Figure 5 – lane 2). These results were repeated at least two times. One isolate generated pMAB01 amplicons with all primer pairs but did not show the ∼50-kb fragment in the PFGE-*Dra*I analysis. A hybridization band was detected near the origin of the gel (Figure 5 – lane 3). In a second experiment using the same isolate, two hybridization bands were detected by Southern blot hybridization (Figure 5 – lane 4). The last two epidemic isolates did not show any evidence of the presence of pMAB01 sequences using PCR, PFGE or Southern blot hybridization (Figure 5 – lane 5). The PCR analysis was repeated with different DNA extractions on different days, and the results were consistently negative, confirming that these two surgical isolates do not carry the pMAB01 plasmid.

The presence of pMAB01 sequences was evaluated using PCR with five primer pairs (*trfA, trbE, qacEdelta1, oriT* and *merE*), and 11 isolates from sputum, bronchoalveolar lavage or urine [28] that were not connected to the epidemic, but showed PFGE patterns that were indistinguishable or closely related to the PFGE patterns of the surgical outbreak isolates. The pMAB01 amplicons were only detected in three isolates, and the plasmid was demonstrated by the presence of a ∼50-kb band with PFGE-*Dra*I that hybridized with the *trfA* probe. The remaining 8 isolates from this group, the 13 isolates showing unrelated PFGE patterns and the two reference strains did not show any evidence of the presence of pMAB01 sequences (Table 2).

In conclusion, 13 of the 15 surgical isolates and three isolates obtained from sputum, bronchoalveolar lavage and urine generated plasmid amplicons. The remaining 25 isolates did not show any evidence of the presence of this plasmid.

### Plasmid stability

Ten in vitro passages of three surgical outbreak isolates bearing the plasmid pMAB01 were performed in liquid medium in a period of two weeks. The analysis of 100 colonies from the 10^th^ passage plate of each isolate by amplification of *trfA1* gene from pMAB01 by PCR (PCR-*trfA*) showed that the plasmid was stably maintained in isolates B52 [1] and IAL 042 [29], while 2% of the INCQS 00594 isolate’s colonies lost the plasmid. Plasmid cure was demonstrated by PFGE-DraI (Figure 5 – lane 6).

### pMAB01 transfer to Mycobacterium smegmatis and Escherichia coli

Mating experiments using the INCQS 00594 strain as donor and *M. smegmatis* mc^2^155, *E. coli* C600 Nal^R^ or *E*. *coli* BL21(DE3) as recipients were performed on solid support (two experiments with 5 and 10 days incubation) and in liquid medium (three experiments with 4, 7 and 10 days incubation). No transconjugants of *M. smegmatis* mc^2^155 or *E. coli* C600 Nal^R^ were detected. BL21(DE3) transconjugants were detected at a frequency of 5.24×10^−8^ transconjugants per recipient in experiments on solid support. Conjugation occurred in the presence of DNase I, confirming that the plasmid passed from one bacterium to the other without being released to the external millieu.

**Figure 6:**
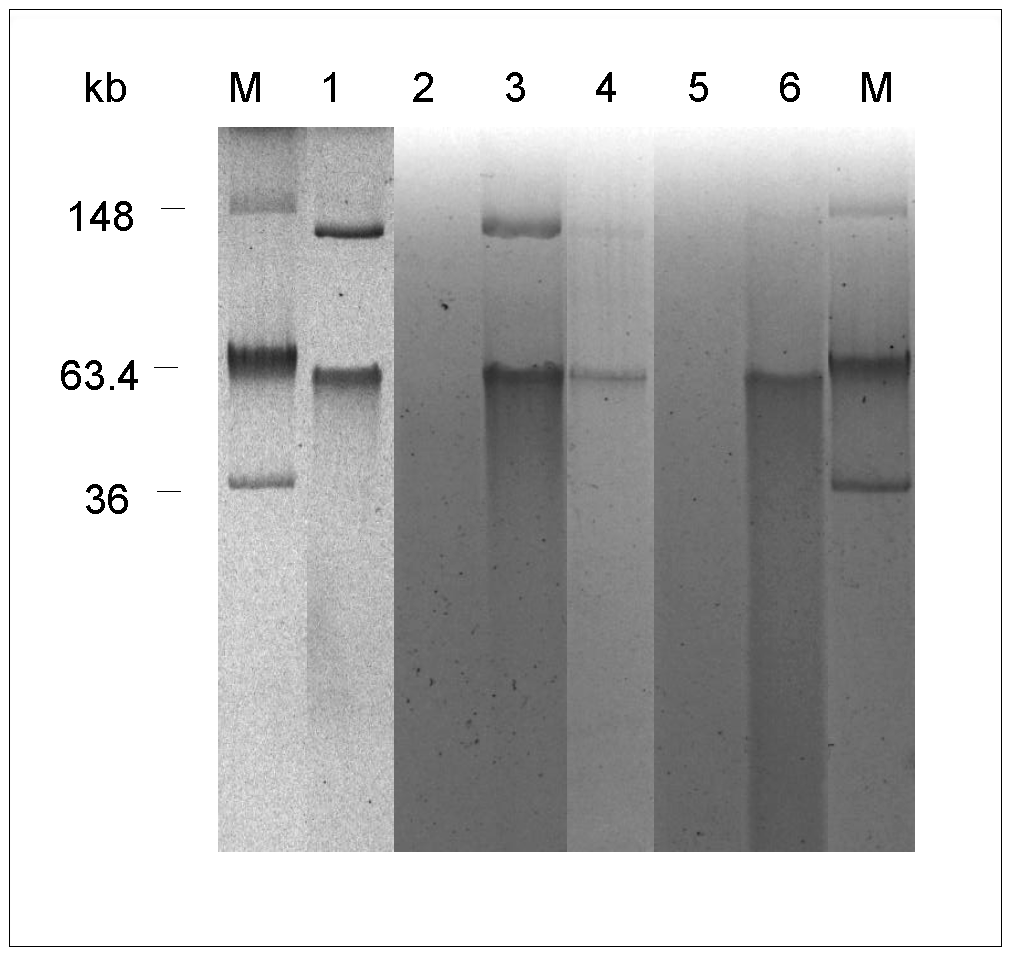
Plasmid pMAB01 purified from INCQS 00594 and from *E.coli* transconjugants and transformants. Plasmid from *M. abscessus* subsp. *bolletii* INCQS 00594 was extracted with QIAGEN Plasmid Maxi Kit and plasmids from *E. coli* with QIAGEN Plasmid Mini Kit. M, *E. coli* NCTC39 R861 (used as marker), 1, *M. abscessus* subsp. *bolletii* INCQS 00594; 2, *E. coli* BL21(DE3); 3, *E. coli* BL21(DE3) transformed with pMAB01; 4, transconjugant *E. coli* BL21(DE3); 5, *E. coli* C600 Nal^R^; and 6, transconjugant *E. coli* C600 Nal^R^

Competent *M. smegmatis* mc^2^155, and the *E. coli* strains DH5α, JM101, LM1035 and BL21(DE3) were electroporated with the purified plasmid pMAB01. Only *E. coli* BL21(DE3) was transformed by pMAB01. The presence of intact pMAB01 in nine transformed colonies was confirmed by PCR of 10 plasmidial genes (Table 1) and by plasmid isolation (Figure 6). The transformants of *E. coli* BL21(DE3) were named BL21(DE3)pMAB01.

One colony of BL21(DE3)pMAB01 was used in a second round of mating experiments. pMAB01 was transferred from *E. coli* BL21(DE3)pMAB01 to *E. coli* C600 Nal^R^ at frequency of 1.14×10^−6^ transconjugants per recipient after mating experiments on solid support with DNase I. The presence of pMAB01 in *E*. *coli* C600 Nal^R^ transconjugants was confirmed by PCR-*trfA1* gene and by plasmid isolation (Figure 6). No transconjugants were detected in mating experiments performed in liquid medium.

## Discussion

The results of this study confirmed the presence of an IncP-1β plasmid in the *M. abscessus* subsp. *bolletii* strain, which was responsible for the nationwide epidemic of surgical-site infections in Brazil. The presence of this plasmid appears to be restricted to this particular strain of *M. abscessus* subsp. *bolletii*, which caused more than 2,000 infections during the epidemic and several other sporadic infections not related to surgical procedures [4], [28]. To date, pMAB01 has not been detected in isolates showing other PFGE patterns.

Consistent with other IncP-1 plasmids, pMAB01 is a circular molecule. *In silico* restriction analysis confirmed the absence of restriction sites for *Dra*I and *Ase*I, the two enzymes used in PFGE experiments, consistent with the hypothesis that the ∼50-kb band (which is visible with PFGE-*Dra*I and PFGE-*Ase*I) corresponds to the circular plasmid. Interestingly, only a faint band of approximately 50 kb was observed when PFGE experiments were performed without DNA digestion (data not shown). This poor visibility of plasmids in undigested PFGE gels was also observed with a linear plasmid of *M. avium* in a previous study [12].

IncP-1 plasmids are the most promiscuous of all known plasmids to date [26]. IncP-1β plasmids have been isolated from municipal wastewater treatment plants in Germany [26], [30], [31], Norwegian soils [24], estuarine waters in Portugal [32], areas of the Nura river in Kazakhstan [33], the soil bacteria *Sphingomonas* sp. A1 [34] and clinically important bacteria, such as *Bordetella pertussis* [35] and *Burkholderia cepacia* [36]. This present study is the first description of the presence of such plasmids in mycobacteria.

Identity matrices, genetic distance calculations and phylogeny analyses suggested that pMAB01 is closely related to plasmids BRA100 (isolated from the biopsy channel of a flexible bronchoscope (unpublished)), pJP4 (isolated from *Ralstonia eutropha* [37]), pAKD33 (isolated from agricultural soil [24]) and pB10 (from wastewater treatment plants in Germany [21]). Because the gene *klcA* showed the largest variability among the analyzed sequences, as shown in Figure 3B, a second tree that excluded *klcA* was generated. Although the analysis of this gene lowered the credibility values at certain nodes, the same topology was obtained from trees with and without *klcA* (data not shown). Alterations in the genetic distances, particularly when considering the *klcA* gene, could result from unequal nucleotide substitution rates or homologous recombination events among taxa, thereby generating distinct evolutionary histories (i.e., different genes in each plasmid might have different ancestors [38]). The results of the Bayesian analysis showed that the archetype IncP-1 plasmid R751 is closely related to pB8 and phylogenetically distant from pKJK5, as previously suggested by Bahl et al. [25].

pMAB01 encodes stabilization systems that can potentially ensure its maintenance and inheritance. The results of plasmid stability testing confirmed that this plasmid was stably maintained after ten passages in liquid medium without selective pressure, as only two out of a total of 300 colonies tested lost the plasmid. Active partitioning involves IncC and KorB proteins, and 11 copies of the KorB-binding *cis*-acting motif (5′TTTAGCCGCTAAA3′) are dispersed throughout the entire plasmid. The KorA protein is encoded by *incC* using a different reading frame and is thought to regulate genes involved in vegetative replication and stable maintenance [17]. The genes *din* and *relE* represent a toxin-antitoxin system that potentially functions in post-segregational killing and might mediate the exclusion of competing plasmids. These regions are present in plasmid pB10, where the two stabilization systems likely ensure highly stable plasmid maintenance and inheritance, as observed in *Escherichia coli* and other proteobacteria [26], [39]. As a consequence, genetic determinants will rarely be lost, even in the absence of selective pressure.

The plasmid pMAB01 possesses a complete system for conjugative DNA transfer. The gene products encoded by the Tra2 core region have counterparts in bacterial type IV secretion systems that are involved in conjugation and the transfer of virulence factors to eukaryotic cells. The Tra2 gene products encode structural components (TrbC, TrbF) of the sex pilus and pilus biogenesis, channel components (TrbB, D, E, F, G, H, I, J and L) and gene products involved in energy provision for DNA transport and pilus biogenesis (TrbB and TrbE). TrbN is a transglycosylase predicted to catalyze the lysis of the peptidoglycan layer. TrbJ and TrbK are potentially involved in entry exclusion, thus preventing the formation of the DNA entry pore and plasmid uptake into a cell that already harbors an IncP-1 plasmid. TrbA controls the expression of the *tra* and *trb* operons [17], [26].

The Tra1 module contains the specific components TraJ, TraK, TraI and TraH of the relaxosome complex. When the relaxosome complex is assembled at the *cis*-acting origin of transfer *oriT*, which is located in the intergenic region between the divergently transcribed *traJ* and *traK* genes, TraI catalyzes single-strand nicking within the 6-bp *nic* site (5′ATCCTG3′) and becomes covalently bound to the 5’ terminal nucleotide (G) of the nicked strand. Subsequently, TraC-mediated rolling-circle transfer replication is initiated, and TraG guides the relaxosome complex to the mating channel [17], [26].

We confirmed that pMAB01 could be transferred by conjugation from *M. abscessus* subsp. *bolletii* strain INCQS 00594 to *E. coli* at and between two distinct *E. coli* strains, indicating that the pMAB01 transfer modules per se are functional. The plasmid transfer rate between the two *E. coli* strains was higher than the transfer rate from *Mycobacterium* to *E. coli*. Schlüter et al [40] showed similar results with the IncP-1β plasmid pB8. The transfer frequency from *Pseudomonas* sp to *E. coli* was low (10^−6^ to 10^−7^ per recipient), but once established in *E. coli*, this plasmid was transferred at higher frequency between distinct *E. coli* strains (1.6×10^−1^ per recipient).

Conjugal transfer of pMAB01 to *M. smegmatis* mc^2^155 could not be demonstrated here. *M. smegmatis* mc^2^155 lacks a suitable marker for selection of transconjugants. Moreover, it has similar growth rate of *M. abscessus,* used as donor. A total of 120 mycobacterial colonies were randomly picked from the mating plates in five experiments and only one colony was identified as *M. smegmatis.* The remaining were colonies of the INCQS 00594 donor strain, identified by PRA-*hsp65* as *M. abscessus* type 2. However, the PCR of plasmidial genes was negative with this single colony of *M. smegmatis*, meaning that it was not a transconjugant. These results cannot definitively disprove the conjugation between *M. abscessus* and *M. smegmatis,* but if it occurs, the transfer frequency must be low.

Conjugation in mycobacteria has been demonstrated. A conjugation-like process was described in *Mycobacterium smegmatis* in the early 1970s [41]. This unconventional conjugal transfer is dependent on a chromosomally encoded transfer system [42]. The in vitro conjugal transfer of the linear plasmid pMA100 from *Mycobacterium avium* to *Mycobacterium kansasii* and *Mycobacterium bovis* BCG was experimentally verified, although the responsible genes have not been identified [12]. The detection of an IncP-1β promiscuous plasmid in *M. abscessus* subsp. *bolletii* confirmed for the first time that mycobacteria could receive and replicate genetic material from distinct phylogenetic groups of bacteria, possibly through conjugal transfer.

One out of four *E. coli* strains tested could be transformed by pMAB01. But we could not detect the transformation of *M. smegmatis* mc^2^155 by pMAB01 in a series of five experiments performed in different days and with different plasmid and bacterial preparations. *M. smegmatis* mc^2^155 is a high efficiency transformation mutant [43]. Competent *M. smegmatis* was successfully transformed with mycobacteria-*E. coli* shuttle plasmids as pMV261, pMV263 and pFPV27, yielding the expected 10^5^ transformants per microgram of DNA [43] (data not shown). No conclusion could be drawn so far on the lack of transformation of *M. smegmatis* mc^2^155 by plasmid pMAB01.

The two genetic load regions of plasmid pMAB01 carry antimicrobial resistance genes related to kanamycin, streptomycin, sulfonamide and mercury and quaternary ammonium compounds.

The Tn*501*-like *mer*-resistance transposons present in the pMAB01, pB10, R906, pJP4 and pAKD plasmids possess the same insertion and 5-bp direct repeat sequence (5’TGCCT3’) adjacent to the IR, suggesting that these transposons are all derived from a progenitor transposon that entered a common ancestor of these plasmids by transposition insertion [21], [24], [44]. In pMAB01, pB10 and R906, the transposase gene (*tnpA’*) is interrupted at the same point, i.e., after 264 bp, by the insertion of a remnant copy of Tn*5393c*. pMAB01 has a unique deletion in the Tn*5393c tnpR* gene region, which is most likely caused by insertions and deletions of unknown transposable elements. The *tnpR* gene is complete in pB10 and R906. pMAB01 also lacks the 3’ region of the truncated *tnpA* transposase gene and the left IR from the Tn*501*-like *mer* transposon, which are both present in pB10 and R906. An intact copy of IS*1071* is present in pMAB01 and R906. In pB10, a truncated derivative of the tetracycline-resistance transposon Tn*1721* is present in the IS*1071 tnpA* gene (Figure 3a). The occurrence of a Tn*501*-like mercury-resistance transposon in exactly the same site in pMAB01, pB10, R906 and other IncP-1β plasmids, and the high level of sequence identity between the *merA* genes, suggests that all plasmids share a common ancestor that previously contained this transposon and subsequently gained different accessory genes [24].

Mercury resistance can contribute to bacterial survival in diverse aquatic environments [33]. Mercury resistance is common in rapidly growing mycobacteria [45], and mercury resistance plasmids have been detected in *M. abscessus* [46], *Mycobacterium marinum* [16] and *Mycobacterium scrofulaceum* [47]. It is possible that pMAB01, as a multiple resistance plasmid, confers increased resistance to environmental conditions or better adaptation to the human host to the particular strain of *M. abscessus* subsp. *bolletii* that caused the nationwide epidemic in Brazil.

Plasmids pMAB01 and pB10 have a typical class 1 integron with completely conserved 5′ and 3′ segments inserted in the same location between the Tra2 and Tra1 regions (load 2 in Figure 2). Both plasmids have a 25-bp inverted repeat (IR) located 178 bp downstream of the *intI* gene and lack the opposite IR downstream of *orf5* [21]. The genes *ufp31.0* and *parA*, which enhance stable plasmid inheritance via the resolution of multimers, are present in pAKD33 but absent in pMAB01 and pB10 [24]. These observations provide further evidence of the existence of a common ancestor of pMAB01 and pB10. The gene cassettes present in pMAB01 are unique and different from those present in pB10. The first cassette, *aac(6’)-Ib*, encodes an aminoglycoside 6'-N-acetyltransferase that confers resistance to tobramycin, kanamycin and other aminoglycosides and is identical to the *aac(6’)-Ib* gene present in *Klebsiella pneumoniae* plasmid pY2 (accession number AF227505) and *Enterobacter cloacae* plasmid pUL3AT (accession number YP_005473814). The second cassette encodes a hypothetical protein with a glyoxalase-like domain and has been identified in a variety of structurally related metalloproteins, including the type I extradiol dioxygenases, glyoxalase I and a group of antibiotic resistance proteins.

QacEdelta1 belongs to a family of proteins that can export different drugs and biocides. The role of this protein in the reported resistance to glutaraldehyde of outbreak isolates [3], [9] has not been demonstrated. Moreover, among the two outbreak isolates used to illustrate resistance to high glutaraldehyde concentrations (MIC  =  8%), one contains the pMAB01 plasmid and the other does not [9]. Therefore, other genes involved in glutaraldehyde resistance remain to be identified.

It is known that the migration of circular DNA relative to linear DNA is not constant, and the electrophoresis of large circular DNA is complicated by the tendency of relaxed circles to become trapped in agarose [48]. There is evidence that nicked plasmids can remain fixed at the origin of PFGE gels [49], and this effect could explain the localization of the *trfA* hybridization band near the origin in lane 3 of the gel shown in Figure 5. Among the 152 isolates obtained from surgical patients [4], 18.4% lacked the ∼50-kb band in the PFGE analysis. The variable migration of circular plasmids could partially explain the lack of visualization of the ∼50-kb band.

PCR and Southern blot hybridization experiments conclusively demonstrated that two surgical isolates lacked the pMAB01 plasmid. These isolates showed the characteristic outbreak PFGE pattern without the ∼50-kb band. Thus, it was proposed that the initial outbreak strain carried this plasmid and that it was subsequently lost under certain circumstances, such as the propagation of the epidemic from 2004 to 2008 or by spontaneous plasmid curing during several passages in culture. In support of this hypothesis, a 2% plasmid cure frequency was demonstrated in one outbreak isolate.

This report presents the first description of the presence of an IncP-1β plasmid in mycobacteria. This finding demonstrates that mycobacteria naturally undergo genetic exchange in the environment. The acquisition of broad-host-range plasmids by ubiquitous mycobacteria could potentially generate specific strains that might be better adapted to causing human disease. Therefore, the role of this plasmid in the generation of outbreaks caused by environmental mycobacteria requires further investigation.

## Materials and Methods

### Mycobacterial and *E. coli* isolates, culture and DNA extraction

Fifteen *M. abscessus* subsp. *bolletii* strains isolated from the epidemic of surgical-site infections, including the collection strain INCQS 00594 and 24 isolates from patients not connected to the epidemic were included in this study. These isolates were randomly selected from our *M. abscessus* subsp. *bolletii* collection, which comprises 168 isolates from the epidemic of surgical-site infections and 110 isolates from sputum, bronchoalveolar lavage, urine or blood from patients not connected to the epidemic. All of the isolates were provided by the acknowledged collaborators and were described in previous publications [1], [3], [4], [28], [50]. Type strains *M. abscessus* ^T^ ATCC 19977 and *M. massiliense* ^T^ CCUG 48898 were included for comparison. *M. smegmatis* mc^2^155 [43] and *E. coli* strains BL21(DE3), DH5α, JM101, LM1035 [51] and a spontaneous mutant of *E. coli* C600 [51] showing nalidixic acid resistance (C600Nal^R^) were used in conjugation and transformation experiments.

The mycobacterial isolates were cultivated in Middlebrook liquid 7H9 media or solid 7H10 media (Becton Dickinson, Franklin Lakes, NJ) supplemented with oleic acid, albumin, dextrose and catalase (OADC; Becton Dickinson). The cell lysates were obtained through repeated cycles of freezing and thawing and were subjected to PCR analysis.

Strain INCQS 00594 was cultivated in Mueller-Hinton liquid medium supplemented with Tween 80 (final concentration of 0.1%) until an OD_600_ of ∼0.6–0.8 was reached. The extraction and purification of DNA were performed as previously described, with modifications [52]. Briefly, bacteria were incubated with D-cycloserine for 48 h and lysed with lysozyme (5 mg/mL), proteinase K (1 mg/mL) and 3% sodium dodecyl sulfate (SDS). The DNA was extracted with phenol/chloroform/isoamyl alcohol (25∶24∶1), precipitated with 0.6 volumes of isopropanol and resuspended in 1X buffer TE (10 mM Tris-HCl, pH 7.5, 1 mM EDTA). The DNA purity was evaluated by measuring the UV absorbance at 230, 260 and 280 nm.

*E. coli* was cultivated in Luria Bertani medium (LB) (10 g of Bacto tryptone, 5 g of yeast extract, and 10 g of NaCl in 1 liter of distilled water) or LB agar plates. Antibiotics used for selection of transconjugants and transformants were nalidixic acid (Sigma Chemical Co., St Louis, Mo.) 30 µg/mL and kanamycin sulfate (Life Technologies, Grand Island, NY, USA) 50 µg/mL.

### Sequencing, assembly and annotation of pMAB01

The prototype outbreak isolate INCQS 00594 was sequenced using the SOLiD V3 platform (Life Technologies Co., Carlsbad, CA) with a mate pair library of 50-bp fragments. The obtained reads were subjected to quality filtering using the Quality Assessment software [53], and reads showing an average Phred quality of less than 20 were eliminated. The filtered reads were subjected to *ab initio* assembly using the CLC Genomics Workbench software with an 80% minimum similarity and a minimum alignment length of 35 bp. After assembly, the generated contigs were mapped against the NT databank from NCBI (http://www.ncbi.nlm.nih.gov); only alignments of more than 50 bp and *e-values* of less than 1×10^5^ were used to identify the closest organisms for mapping the filtered reads. The reference plasmid was identified and used to map the short reads with a minimum 70% similarity and 80% contig size to generate a plasmid draft sequence with gaps. Gap closure was achieved using short read recursive alignments [54] against the plasmid pB10 genome.

To resolve low confiability regions, the Sanger sequencing method was employed using INCQS DNA and primers derived from the obtained sequence and plasmid pB10 (accession number: NC_004840) (Table 1). The final sequence was automatically annotated using the RAST software [55] followed by manual curation using the Artemis program [56]. The base composition of the genome and of each CDS was obtained using Geecee from the EMBOSS package, which calculates G+C percentages.

### Genetic distance and phylogenetic analysis

Genetic distance matrices were generated using the Gegenees program [57] by comparing the complete nucleotide sequence of pMAB01 and 18 representatives of different subgroups of IncP-1 plasmids deposited in the NT databank from the NCBI.

For the genetic distance and phylogenetic analyses, nucleotide and amino acid sequences of genes *trfA, ssb, korB* and *klcA* were selected. The sequences from each plasmid were concatenated and aligned using ClustalW [58] with the default parameters. Minor modifications of the alignment were manually performed using BioEdit v. 7.0.9.0 [59].

The genetic distances were estimated for the nucleotide and amino acid sequences using the maximum likelihood and p-distance method with the MEGA v. 5.1 program [60]. The phylogenetic reconstructions were performed using Bayesian analysis with nucleotide sequences. Gaps were treated as missing data in all analyses. Kakuzan v. 4, a script written in the Perl language, was selected as the most appropriate model for Bayesian analysis using the BIC criterion [61]. The Bayesian tree was reconstructed using MrBayes v. 3.2.1 [62] with the HKY + gamma distribution model for partitions *trfA*, K80 + Gamma for *ssb* and GTR + gamma for *korB* and *klcA*. Trace plot values were used to confirm the convergence of the MCMC. Metropolis-coupled Markov chain Monte Carlo (MCMCMC) sampling was conducted with four chains run for 4,000,000 generations, using the default model parameters as starting values. All trees were rendered using FigTree v. 1.3.1 (http://tree.bio.ed.ac.uk/software/figtree/).

### Detection of plasmid sequences in *M. abscessus* subsp. *bolletii* isolates

Ten different regions of the pMAB01 plasmid were amplified using the primers shown in Table 1. These regions contain genes related to plasmid replication, conjugative function and resistance to antimicrobials and mercury. The PCR reactions contained 15 mM Tris-HCl pH 8.75, 50 mM KCl, 0.1% Triton X-100, 2 mM MgCl_2,_ 140 µM dNTPs, 0.4 µM of each primer, 0.3 U of *Taq* DNA polymerase (RBC Biosciences Corp., Taipei, Taiwan) and 2 µL of bacterial lysate in a final reaction volume of 15 µL. The PCR conditions were performed as follows: initial denaturation at 95°C for 3 min followed by 35 cycles at 95°C for 30 s, 65°C for 30 s and 72°C for 1 min and a final extension at 72°C for 5 min. The annealing temperature in the *kleE* and *traE* amplifications was 60°C.

The PFGE analysis with undigested or *Dra*I-digested DNA was performed as previously described [1], [28]. PFGE gels were photographed, and the DNA was blotted onto nylon membranes (Hybond-N-plus; GE Healthcare, Little Chalfont, UK) and hybridized with a probe complementary to the *trfA* gene. The probe was prepared using PCR with the primers described in Table 1 and DNA from isolate INCQS 00594. The probe was purified using the QIAquick PCR Purification kit (Qiagen, Valencia, CA). The probes were labeled with [α-32^P^]dCTP using Ready-To-Go DNA Labeling Beads (GE Healthcare) according to the manufacturer’s instructions.

Hybridizations were performed at 55°C using the ECL Hybridization buffer (ECL kit RPN3000, GE Healthcare). After 16 h of incubation, the membranes were washed with SSC 2X (20X SSC buffer contains 300 mM sodium citrate and 3 M NaCl) and SDS 0.1% at room temperature for 15 min and with SSC 1X plus SDS 0.1% at 55°C for 15 min. The membranes were exposed to Hybond X-ray film (GE Healthcare) at –80°C for 24 hours.

### Plasmid stability testing

The stability of pMAB01 plasmid was tested using three isolates, INCQS 00594, B52 [1] and IAL 042 [29], according to the protocol described by da Silva Rabello et al. [63] with minor modifications. A single colony of each isolate was cultivated in 10 ml of LB broth, at 37°C, in a shaker, at 180 rpm, until the optical density at 600 nm reached 0.6 to 0.8. The culture was diluted 1∶10 with fresh LB broth, and was incubated under the same conditions. After ten passages, serial dilutions were plated on LB agar, and 100 random colonies were transferred to a new plate. The presence of pMAB01 in these colonies and in the original colonies was verified by PCR-*trfA1* as described above. Plasmid cure was confirmed by PFGE-DraI.

### pMAB01 plasmid DNA isolation

*M. abscessus* subsp. *bolletii* INCQS 00594 strain was cultivated in 1.2 L of LB liquid medium supplemented with kanamycin 50 µg/ml (LB-Km). The bacterial pellet was resuspended in 1X buffer TE (25 mL) with lysozyme (10 mg/mL) and proteinase K (200 µg/mL) and incubated in a shaker at 37°C, 180 rpm, for 24 hours. Plasmid isolation was carried out using QIAGEN Plasmid Maxi Kit (QIAGEN, Valencia, CA).

pMAB01 was purified from *E. coli* BL21(DE3)pMAB01 and from *E. coli* transconjugants using QIAGEN Plasmid Mini Kit (QIAGEN).

### Bacterial transformation by pMAB01

Competent *M. smegmatis* mc^2^155 and *E. coli* DH5α, JM101, LM1035 and BL21(DE3) strains were electroporated with 100 ng of pMAB01 plasmid DNA in a Gene Pulser Xcell™ Electroporation System using a 0.2 cm Gene Pulser cuvette (Bio-Rad Laboratories Inc., Hercules, CA). Electroporation conditions for *E. coli* strains were: 200 Ω resistance, 25 µF capacitance and 2.5 kV voltage. With *M. smegmatis*, the resistance was changed to 1,000 Ω. After electroporation, *E. coli* strains were incubated with shaking in 1 mL of LB liquid medium at 37°C for at least one hour before plating onto LB-Km agar plates. *M. smegmatis* was incubated in one mL of 7H9-ADC for two hours before plating on LB-Km.

### Mating experiments

The INCQS 00594 isolate and *E.coli* BL21(DE3)pMAB01 [*E. coli* BL21(DE3) strain transformed with pMAB01 plasmid by electroporation] were used as donors of pMAB01 plasmid. *E. coli* C600 Nal^R^ and BL21(DE3), and *M. smegmatis* mc^2^155 were used as recipient strains. Mycobacteria were cultivated in LB broth for two days and *E. coli* strains for 16h before the mating experiments.

#### Mating experiments on solid support

Aliquots of 500 µL of each culture were centrifuged (12,000 g) and washed once with fresh LB broth. The resuspended pellets of each pair of donor and recipient were mixed and carefully deposited on sterile 0.45 mm membranes (Millipore, Billerica, MA), which were incubated over LB agar plates for five or ten days. The mating experiments were done in duplicate, with or without adding 75 U of deoxyribonuclease I (DNase I) (LifeTechnologies, Carlsbad, CA, USA) to the culture mix. After each incubation period, the filters were washed with 1 ml of LB broth and bacteria were transferred to a microtube. Serial dilutions were plated on LB-Km. When *E. coli* C600 Nal^R^ was used as recipient, LB-Km was supplemented with nalidixic acid 30 µg/ml (Sigma-Aldrich Corp., St. Louis, MO) (LB-Km-Nal).

#### Mating experiments in liquid medium

Aliquots of 1 ml of cultures of INCQS 00594 and 100 µl of *E*. *coli* BL21(DE3) or C600 Nal^R^ were centrifuged (12,000 g), washed once with LB broth, resuspended in 1 ml fresh LB broth and mixed in disposable conical tubes. The volume was completed to 3 mL and the tubes were incubated at 37°C without shaking. Aliquots of 100 µl were retrieved from each tube at days 4, 7 and 10 and serial dilutions were plated on LB-Km or LB-Km-Nal. Mating experiments of *E. coli* BL21(DE3)pMAB01 and *E. coli* C600 Nal^r^ were done using aliquots of 1 mL of each culture. The mixture was incubated at 37°C for 24 hours and plated on LB-Km-Nal.

*E. coli* BL21(DE3) transconjugants were detected by growth on LB-Km and C600 Nal^R^ transconjugants were screened on LB-Km-Nal plates after 24-48h incubation at 37°C. *M. smegmatis* transconjugants were screened by PRA-*hsp65* and PCR of plasmid genes among approximately 20–25 isolated colonies randomly picked in each experiment.

After the mating experiments, transconjugants, recipients and donors were counted on LB, LB-Km or LB-Km-Nal plates.

### Nucleotide sequence accession number

The complete pMAB01 sequence obtained in this work was deposited in GenBank under accession number CP003376.
