# Correction: The Detection and Sequencing of a Broad-Host-Range Conjugative IncP-1β Plasmid in an Epidemic Strain of *Mycobacterium abscessus* subsp. *bolletii*

**DOI:** 10.1371/annotation/5dd55ed1-2fb6-4672-9142-fb01331567e1

**Published:** 2013-09-24

**Authors:** Sylvia Cardoso Leão, Cristianne Kayoko Matsumoto, Adriana Carneiro, Rommel Thiago Ramos, Christiane Lourenço Nogueira, James Daltro Lima Junior, Karla Valéria Lima, Maria Luiza Lopes, Horacio Schneider, Vasco Ariston Azevedo, Artur da Costa da Silva

There was an error in the plasmid size reported in the article. The article refers to a 56,264-bp plasmid which is incorrect; the correct size is 56,267-bp, as specified in the GenBank record CP003376: http://www.ncbi.nlm.nih.gov/nuccore/CP003376.1. The authors apologize for any inconvenience or confusion this may have caused to readers.

The phylogenetic analysis reported in the article made use of IncP-1 plasmid sequences identified in GenBank. The relevant GenBank accession numbers of the plasmid sequences used for the genetic distance and phylogenetic analysis are provided below:

plasmid

GenBank accession number

pMAB01

CP003376

BRA100

CP003505

pA81

CP002288

pAKD1

JN106164

pAKD16

JN106167

pAKD33

JN106174

pB3

AJ639924

pB4

AJ431260

pB8

AJ863570

pB10

AJ564903

pHH3414

JQ004408

pJP4

AY365053

pKJK5

AM261282

pTB11

AJ744860

pTP6

AM048832

pUO1

AB063332

QKH54

AM157767

pR751

U67194

pBP136

AB237782

The authors commit to making all the sequences used in this article available for research purposes upon request. 

